# Inflammation-associated regulation of RGS in astrocytes and putative implication in neuropathic pain

**DOI:** 10.1186/s12974-017-0971-x

**Published:** 2017-10-27

**Authors:** Pierre J. Doyen, Maxime Vergouts, Amandine Pochet, Nathalie Desmet, Sabien van Neerven, Gary Brook, Emmanuel Hermans

**Affiliations:** 1 Neuropharmacology, Institute of Neuroscience, Université Catholique de Louvain, Avenue Hippocrate B1.54.10, 1200 Brussels, Belgium; 2 Institute for Neuropathology, University Hospital, RWTH Aachen University, Aachen, Germany

**Keywords:** Astrocytes, Glial cells, Neuropathic pain, Inflammation, Regulator of G-protein signaling, Cytokines, Growth factors, Spared nerve injury

## Abstract

**Background:**

Regulators of G-protein signaling (RGS) are major physiological modulators of G-protein-coupled receptors (GPCR) signaling. Several GPCRs expressed in both neurons and astrocytes participate in the central control of pain processing, and the reduced efficacy of analgesics in neuropathic pain conditions may rely on alterations in RGS function. The expression and the regulation of RGS in astrocytes is poorly documented, and we herein hypothesized that neuroinflammation which is commonly observed in neuropathic pain could influence RGS expression in astrocytes.

**Methods:**

In a validated model of neuropathic pain, the spared nerve injury (SNI), the regulation of RGS2, RGS3, RGS4, and RGS7 messenger RNA (mRNA) was examined up to 3 weeks after the lesion. Changes in the expression of the same RGS were also studied in cultured astrocytes exposed to defined activation protocols or to inflammatory cytokines.

**Results:**

We evidenced a differential regulation of these RGS in the lumbar spinal cord of animals undergoing SNI. In particular, RGS3 appeared upregulated at early stages after the lesion whereas expression of RGS2 and RGS4 was decreased at later stages. Decrease in RGS7 expression was already observed after 3 days and outlasted until 21 days after the lesion. In cultured astrocytes, we observed that changes in the culture conditions distinctly influenced the constitutive expression of these RGS. Also, brief exposures (4 to 8 h) to either interleukin-1β, interleukin-6, or tumor necrosis factor α caused rapid changes in the mRNA levels of the RGS, which however did not strictly recapitulate the regulations observed in the spinal cord of lesioned animals. Longer exposure (48 h) to inflammatory cytokines barely influenced RGS expression, confirming the rapid but transient regulation of these cell signaling modulators.

**Conclusion:**

Changes in the environment of astrocytes mimicking the inflammation observed in the model of neuropathic pain can affect RGS expression. Considering the role of astrocytes in the onset and progression of neuropathic pain, we propose that the inflammation-mediated modulation of RGS in astrocytes constitutes an adaptive mechanism in a context of neuroinflammation and may participate in the regulation of nociception.

## Background

Astrocytes express a variety of G-protein-coupled receptors (GPCRs) that are recognized and activated by several neurotransmitters released from nerve terminals [1]. In response to neuronal signals, these receptors contribute to the physiological regulation of astrocytic functions [2, 3]. GPCRs also play a role in contexts of nervous insults as they influence both the supporting and deleterious properties of reactive astrocytes on nervous recovery [2] and modulate the synaptic activity and plasticity [3, 4]. In neuropathic pain, a chronic pain state subsequent to a nervous lesion, modifications in spinal synapses and activation of astrocytes contribute to central sensitization, a plastic adaptation that leads to the differential processing of nociceptive signals [5]. Hence, the role of astrocytes in the development and maintenance of central pathological alterations is well documented [6, 7]. Activation of glial cells and interaction between glia and neurons have emerged as key mechanisms of neuropathic pain, characterized especially by the synthesis and release of glial mediators such as growth factors and inflammatory cytokines that can activate astrocytes themselves, modulate synaptic activity, and further modulate pain sensitivity [8, 9]. Accordingly, inhibition of astrocytes proliferation and inflammation was shown to reduce hypersensitivity associated behavior in animal models of neuropathic pain [10, 11].

Spinal GPCRs are the primary molecular targets for several endogenous modulators of the nociceptive transmission. In particular, GABAergic, noradrenergic, serotoninergic, cannabinoid, and opioid receptors are densely expressed in the dorsal spinal cord where they inhibit the transmission of pain signals to supraspinal structures [12, 13]. In contrast, other members such as group I metabotropic glutamate receptors present a more complex profile, promoting central sensitization when activated in ascending nociceptive pathways or playing an antinociceptive role when activated in descending pathways [14]. Pharmacological modulators of these receptors are considered as putative tools for clinical pain relief [5, 15]. Consistent with such diversity, neuropathic pain is thought to result from an unbalance between pro- and antinociceptive pathways in the dorsal spinal cord [16]. As the synaptic activity and plasticity can be influenced upon activation of GPCRs on astrocytes [3, 4], regulation of their signaling could contribute to the maintenance of long-lasting neuropathic pain.

Several studies have examined the regulation of GPCRs in neuropathic pain or in model of neuroinflammation [12, 17, 18]. In this line, their associated signaling partners have recently received increasing attention as these proteins physiologically contribute to a fine-tuning of the G-protein cascade [19, 20]. Among them, regulators of G-protein signaling (RGS) are commonly viewed as negative modulators of GPCR signaling, principally via their “GTPase activating protein” function [21]. However, these proteins may also facilitate GPCR activation. Thus, they could promote the membrane expression of the receptors and their interaction with other proteins such as GPCRs themselves and effectors such as kinases (JNK, Ask1) or adenylyl cyclase, to establish functional signaling complexes [22, 23]. Among the large family of RGS, some members are expressed in the central nervous system such as RGS2, RGS3, RGS4, and RGS7 [24, 25].

Consistent with their interaction with GPCR controlling pain pathways, in particular opioid and cannabinoid receptors [26, 27], RGS have been implicated in the regulation of inflammation [28] and neuropathic pain [29, 30]. A growing body of evidence indicates that, along the nociceptive neuraxis, RGS influence analgesic systems and a reduced efficacy of both endogenous pain modulators and exogenously administered therapeutic agents may result from alterations in RGS [26, 31, 32]. Although regulation of RGS is already documented in brain and spinal cord samples [26, 31] or directly within sensory neurons [29], little is known regarding their modulation and expression in glial cells. We first analyzed spinal RGS expression after a spared nerve injury (SNI), a model of neuropathic pain causing robust central inflammatory response [33], and identified them as potential targets to be modified in neuroinflammation. Because neuropathic pain presents a wide complexity, with different cellular and molecular actors all leading together to pain symptoms, we further studied RGS in a more simplified model. The essential role played by astrocytes in central sensitization and neuroinflammation [7, 34], as described earlier, led us to study the regulation of selected RGS in reactive astrocytes and in response to inflammatory mediators.

## Methods

### Spared nerve injury

Female Sprague Dawley rats, 10–12 weeks old, from the institutional animal facility, were used in strict adherence to the EU directive of 22/09/2010 (2010/63/EU). The animals were kept in groups of 2–3 animals per standard makrolon cage with ad libitum access to food at a regular 12:12-h light-dark cycle. The SNI was performed following the model developed by Descosterd with little modifications [35]. Sciatic nerve was exposed on either the left or the right side under sevoflurane anesthesia (5% then maintained at 3% in oxygen). Then, the three peripheral nerve branches of the sciatic nerve (i.e., tibial, common peroneal, and sural nerve branches) were exposed through blunt dissection and freed from the surrounding connective tissue. The animals were at random divided into two groups for SNI and sham surgery, respectively. For SNI, the tibial and common peroneal nerve branches were injured. Injury was inflicted using a non-serrated nerve clamp, i.e., the De Beer clamp (Honer Medizin-Technik GmbH & Co.) exerting a force of 54 N over a period of 30 s [36]. The sural nerve branch was left intact (spared). For the sham surgery, skin incision was made and the sciatic nerve branches were also freed from their connective tissue but were not crushed. Then, the wounds were closed using 4/0 prolene sutures, and the animals were returned to their cage. Postoperative care did not include pain medication as this might interfere with the primary study outcome, i.e., the study of biochemical changes occurring in a context of neuropathic pain.

### Algesimetry

In order to assess mechanical hypersensitivity, the mechanical paw withdrawal threshold (PWT) was determined using the von Frey hair filament test, according to the up-down method [37]. After habituation, the animals were placed in transparent plastic chambers, positioned on an elevated wire mesh. Acclimatization was allowed for a period of about 20 min after which the von Frey test was performed. Herein, a set of eight calibrated von Frey hair filaments (Stoelting) was used: 0.4, 0.7, 1.2, 2.0, 3.6, 5.5, 8.5, 15.1 g. Only the sural nerve territory at the glabrous plantar hind paw surface was stimulated throughout the experiment as this territory remains innervated in injured animals, thus allowing for the assessment of stimulus-response behaviors. Filaments were applied perpendicular to the plantar hind paw surface and maintained in a slightly buckled position for a maximum duration of 8 s, starting with the 2-g filament. The choice for the following filament was based on the response to the previous filament application, being the closest-lower filament in case of a positive withdrawal response (“x”) or the closest-higher filament in case of a negative withdrawal response (“o”). A positive withdrawal response was defined by a brisk paw withdrawal sometimes associated with aversive behavior, such as keeping the stimulated paw elevated, shaking, and/or licking of the paw. After a sequence of six filament application starting either with “o-x” or with “x,” the 50% PWT was calculated as described previously [37]. In case of merely positive or merely negative responses to any filament, cutoff values were assigned (0.4 and 15.1 g, respectively).

### Animal dissection

A total of 36 rats were used for tissue analysis. Eighteen of them underwent a SNI surgery while the others received sham surgery. In each group, four or five animals were sacrificed at 3, 5, 7, 14, and 21 days after the surgery. The spinal cord was extracted by flushing into the spine with phosphate-buffered saline (PBS). Then, the lumbar spinal cord segment was dissected, and the ipsilateral dorsal quadrant was used for further experiments. Tissue samples were frozen at −80 °C to be used for further experiments.

### Astrocyte cultures

At postnatal day 2, the rats were sacrificed and the cortex was isolated by dissection. The hippocampus and meninges were removed, and the cortical gray matter was dissociated in PBS-glucose 0.2%. Astrocytes were then separated from other cells through a Percoll 30% gradient (GE Healthcare). The cells were finally washed in PBS-glucose and seeded in a gelatin-coated 175-cm^2^ flask. Astrocytes were left to proliferate at 37 °C in a humidified atmosphere containing 5% CO_2_ in Dulbecco’s modified Eagle’s medium (glutaMAX, Thermofisher Scientific) supplemented with 10% fetal bovine serum (FBS) (Thermofisher Scientific), 50 mg/mL penicillin–streptomycin (Thermofisher Scientific), and 50 mg/mL fungizone (Thermofisher Scientific) for 2 weeks. Medium was renewed after 1 week. At day 15, trypsinization was performed and cells were transferred in multi-well plates for 2 days in medium supplemented with 10% of FBS. At day 17, serum concentration was decreased to 3% FBS, and when indicated, the medium was supplemented with N^6^,2′-O-dibutyryladenosine 3′,5′-cyclic monophosphate (dBcAMP) (Sigma-Aldrich) or the growth factor cocktail G5 (ThermoFisher Scientific). For some experiments, the inflammatory cytokines interleukin-1 beta (IL-1β) (Bio-Rad Laboratories), interleukin-6 (IL-6) (Bio-Rad Laboratories), and tumor necrosis factor α (TNFα) (Bio-Rad Laboratories) were added to the medium during the last 4, 8, 24, or 48 h of culture at a final concentration of 2, 10, or 50 ng/mL. For all the experiments, the cells were harvested at day 24 and used for further analyses.

### Immunocytochemistry

Astrocytes were seeded in a 24-well plate. After 7 days of maturation in FBS 3%-medium supplemented or not with dBcAMP or the G5 supplement, the cells were washed three times in PBS. Then, they were fixed with paraformaldehyde 4% in PBS for 30 min on ice. After further washing, the cells were permeabilized with Triton X-100 1% (Pharmacia) in PBS. Blocking was made with bovine serum albumin 1% in PBS, and the cells were incubated with mouse monoclonal anti-glial fibrillary acidic protein (GFAP) antibody coupled to Cy3 (1/500, Sigma-Aldrich), overnight at 4 °C. Finally, the cells were exposed to 4′,6-diamidino-2-phenylindole (DAPI) at 0.2 μg/mL (Sigma-Aldrich) in PBS to stain the nuclei. Fluoprep (bioMérieux SA) was used as mounting medium, and the cells were analyzed using an Evos FL Digital Inverted Microscope (Westburg).

### RNA extraction and qPCR

Total RNA was extracted, isolated, and purified with the E.Z.N.A® Total RNA Kit I (Omega Bio-tek, VWR) and reverse transcribed with the iScript cDNA synthesis kit (Bio-Rad Laboratories). qPCR amplifications were carried out using the Bio-Rad CFX Connect™ real-time PCR detection system (Bio-Rad Laboratories), in a total volume of 20 μL containing 10 ng of cDNA template, iTaq Universal SYBR Green Supermix (Bio-Rad Laboratories), and final concentration of 0.5 μM of each primer. Quantitative analysis was performed using delta-delta Ct method, normalized to a housekeeping gene (glyceraldehyde 3-phosphate dehydrogenase (GAPDH)) expression. Melting curve was performed to assess the amplification of a single product, which was then confirmed by a single band migration at the expected size with agarose gel electrophoresis (not shown). The sequences of primers used in this study are indicated in Table 1.

**Table 1:** Primer sequences used in qPCR (5′->3′)

Primer	Sequence	Expected size of the amplicon (bp)
GAPDH forward	GTCTCCTGTGACTTCAACAG	76
GAPDH reverse	AGTTGTCATTGAGAGCAATGC	
RGS2 forward	TGCCCAAAATATCCAAGAGG	205
RGS2 reverse	CGGGAGACAGAATGGAATGT	
RGS3 forward	GTATCTTCGGGCTCATGGAA	192
RGS3 reverse	TTACTTGTCCCCTCCGTCAC	
RGS4 forward	TTCATCTCTGTGCAGGCAAC	192
RGS4 reverse	GGAAGGATTGGTCAGGTCAA	
RGS7 forward	TCGTCACATGAGAGCTGGAC	158
RGS7 reverse	GACAGTGTCCCTTGGCAAAT	

### Statistical analyses

Data are expressed as the mean ± SEM and the GraphPad Prism 5 software was used to perform all statistical analyses using either a one-way or two-way ANOVA followed by a Dunnett’s or Bonferroni’s multiple comparison or a Student’s *t* test. In all statistical analyses, a value of *p* < 0.05 was defined as significant.

## Results

### Temporal expression of RGS in a model of neuropathic pain

**Fig. 1:**
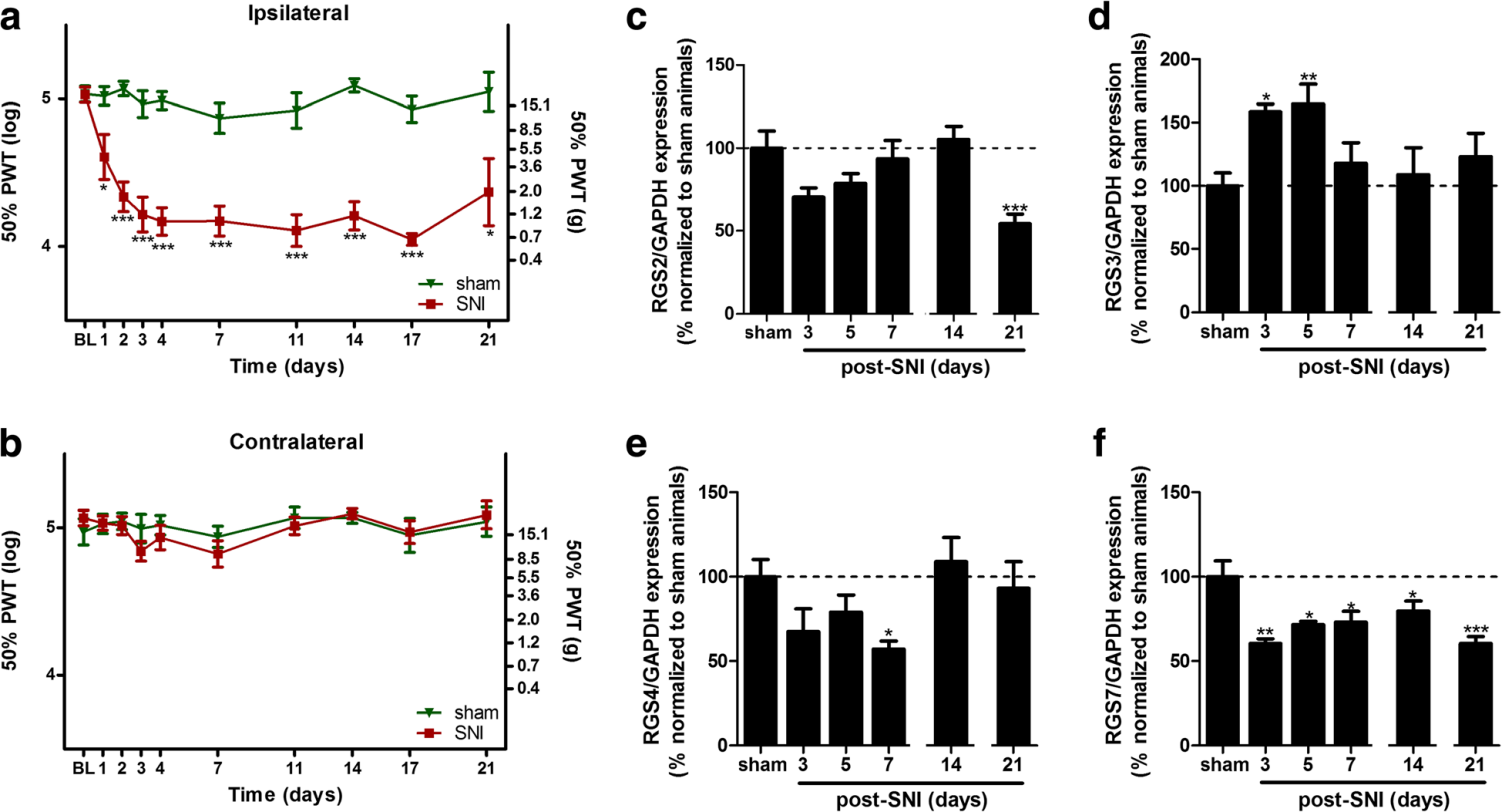
Mechanical hypersensitivity measurement and spinal RGS expression after a SNI. Pain hypersensitivity was determined for the ipsilateral (**a**) and contralateral (**b**) hind paws at baseline (BL; before surgery) and during 3 weeks after sham surgery or SNI. PWT 50% after von Frey filament stimulation was used as a readout for allodynia and calculated as previously described [37]. Data are presented as mean ± SEM; *n* = 10 (0–14 days) or *n* = 5 (14–21 days); two-way ANOVA with Bonferroni’s post test; *p < 0.05, ***p < 0.001 (SNI vs sham). RGS mRNA expression, normalized to GAPDH expression, was then measured in the ipsilateral side of the lumbar spinal cord 3, 5, 7, 14, and 21 days after the SNI lesion and compared to sham-operated animals (**c**–**f**). Data are presented as mean ± SEM; *n* = 4; one-way ANOVA with Dunnett’s post test; *p < 0.05, **p < 0.01, ***p < 0.001 (vs sham). Data shown were obtained from independent experiments either for 3, 5, and 7 days or for 14 and 21 days. Sham, 3, 5, and 7 days: *F*_3,13_ = 2393 (RGS2), 6178 (RGS3), 3434 (RGS4), and 7916 (RGS7). Sham, 14 and 21 days: *F*_2,15_ = 16.83 (RGS2), 0.4426 (RGS3), 0.4370 (RGS4), 17.60 (RGS7)

Putative changes in RGS expression associated with neuropathic pain were examined in the lumbar spinal cord of adult rats subjected to surgical SNI and compared to non-lesioned (sham) animals. SNI resulted in tactile allodynia in the ipsilateral paw persisting for at least 3 weeks after the lesion as assessed by the von Frey filament test (Fig. 1a). No signs in hypersensitivity were observed in the contralateral paw (Fig. 1b). Selected RGS known to be expressed in the CNS [24, 25] were then measured by qPCR in the ipsilateral dorsal quadrant of the lumbar spinal cord of lesioned animals and normalized to the level measured in sham animals, both expressed as relative to GAPDH messenger RNA (mRNA). The nerve lesion was associated with a modest and not significant reduction in RGS2 mRNA until day 21 where it was significantly decreased (Fig. 1c). RGS3 was transiently upregulated, reaching up to 150% of controls, 3 and 5 days after the lesion and returning to a basal level at later stages (Fig. 1d). RGS4 gene expression tended to progressively decrease, with a significant reduction of 40% by 7 days after surgery and returned to basal level overtime (Fig. 1e). Finally, RGS7 was also substantially downregulated, as early as 3 days after lesion, an effect that persisted at all the time points studied (Fig. 1f).

### Regulation of RGS in activated astrocytes

**Fig. 2:**
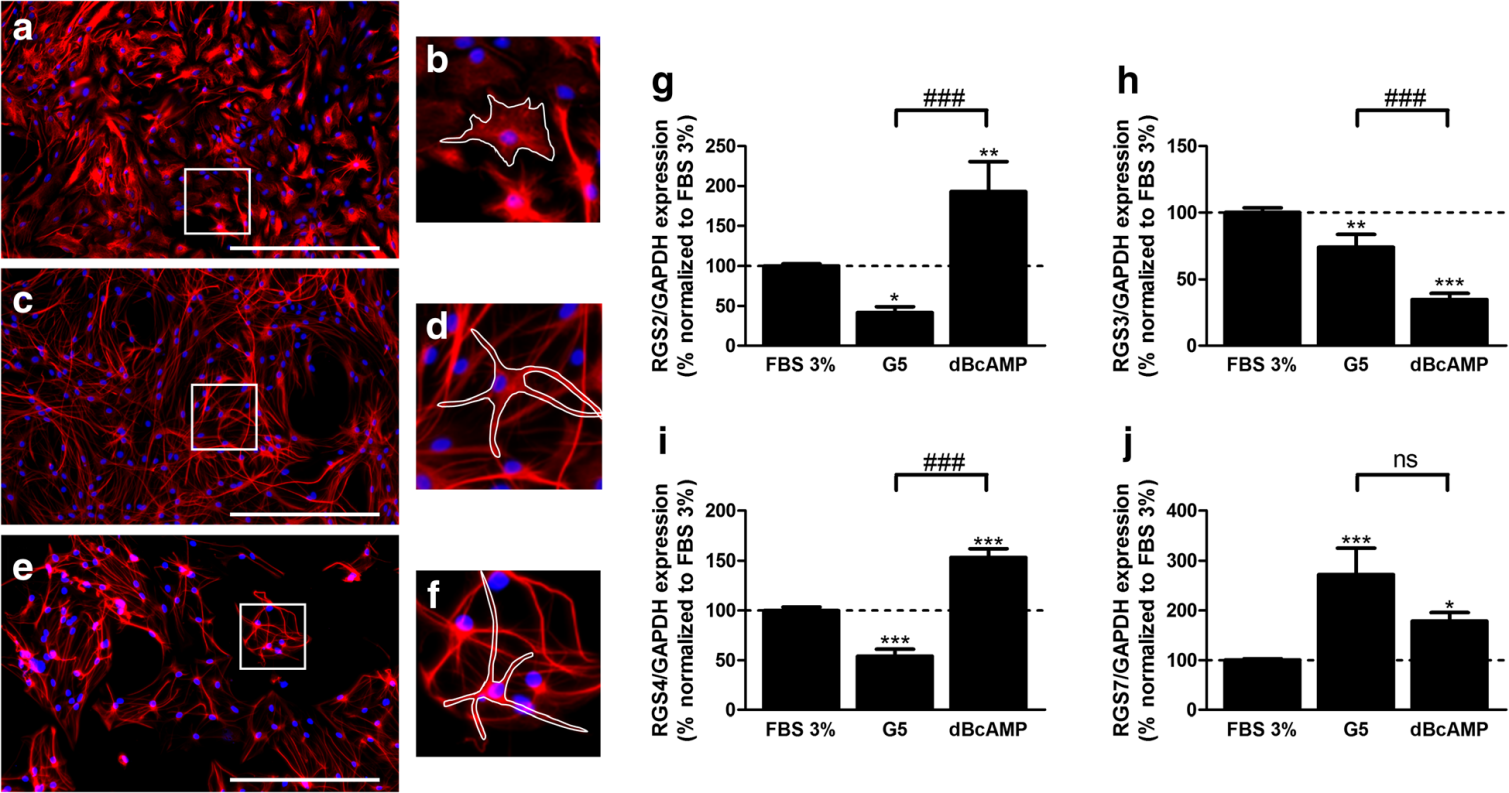
RGS expression and cell morphologies in astrocyte cultures exposed to different maturation conditions. After 17 days in 10% FBS-medium, astrocytes were exposed to FBS 3%-medium alone (**a**, **b**) or complemented either with the G5 supplement (**c**, **d**) or dBcAMP 150 μM (**e**, **f**). Immunostaining was performed using an antibody against GFAP coupled to Cy3 (red) and DAPI staining (blue) was achieved for the nuclei. As highlighted by the white shape, astrocytes adopted a typical protoplasmic (**b**) or stellate (**d**, **f**) morphology following the maturation condition. Bar, 200 μM. For every maturation condition, RGS mRNA expression was then measured by qPCR in independent experiments (**g**–**j**). Expression was normalized to GAPDH expression. Data are presented as mean ± SEM; *n* = 6; ANOVA one-way with Bonferroni’s post test; *p < 0.05, **p < 0.01, ***p < 0.001 (vs FBS 3%), ^###^ *p* < 0.001, ns indicates not significant. *F*_2,22_ = 17.31 (RGS2), 37.70 (RGS3), 56.36 (RGS4), 14.41 (RGS7)

Astrocytes are known to adapt their phenotype to changes in their environment, in particular during nervous insults. These morphological and biochemical changes can be modeled in vitro by exposing cultured astrocytes to defined culture conditions. Accordingly, astrocytes maintained in distinct culture conditions adopted different cell morphologies and spatial organization (Fig. 2a–f). Immunocytochemical detection of GFAP in cultured astrocytes revealed that cells grown in standard conditions (FBS 3%) adopted a typical protoplasmic morphology (Fig. 2b) while cells exposed to a defined growth factor cocktail (G5 supplement) or to dBcAMP (150 mM) for 1 week showed a predominant stellate morphology (Fig. 2d, f). Furthermore, cultures matured in the presence of dBcAMP were organized in clusters (Fig. 2e) while cells grown in the presence of the G5 supplement formed a dense cellular network (Fig. 2c).

The impact of these two culture conditions was examined on the expression of selected RGS by qPCR, and astrocytes maintained in the culture medium containing 3% of FBS were used as control. As shown in Fig. 2g, i, the cells maintained in the presence of the G5 supplement showed a 50% lower expression of both RGS2 and RGS4 as compared to the control. Similarly, RGS3 expression was repressed by 25% (Fig. 2h). In contrast, RGS7 was considerably upregulated in these conditions (up to 250% of the control) (Fig. 2j). The exposure to dBcAMP also profoundly affected RGS expression, but in a distinct pattern as compared to the G5 supplemented condition. Thus, the cells exposed to dBcAMP showed a 1.5- to 2-fold upregulation of RGS2, 4 and 7 compared to standard conditions (Fig. 2g, i, j). At variance, RGS3 was found to be repressed more than 2-fold as compared to standard conditions (Fig. 2h).

### Modification of RGS expression after exposure to inflammatory cytokines

**Fig. 3:**
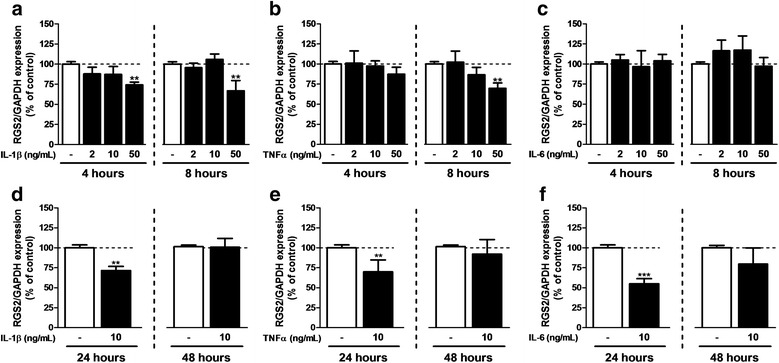
RGS2 expression after exposure to inflammatory cytokines. qPCR was performed on samples after 7 days in FBS 3%-medium in the presence of 2, 10, or 50 ng/mL of IL-1β (**a**, **d**), TNFα (**b**, **e**) or IL-6 (**c**, **f**). Cytokines were added during the 4, 8, 24, or 48 last hours of maturation. RGS2 mRNA expression was normalized to GAPDH. Each experiment was conducted independently. The FBS 3% condition was used as control. Data are presented as mean ± SEM; *n* = 6; ANOVA one-way with Dunnett’s post test; **p < 0.01, ***p < 0.001 (vs control). *F*_3,30_ = 4.799 (IL-1β 4 h), 6.214 (IL-1β 8 h), 0.5637 (TNFα 4 h), 4.517 (TNFα 8 h), 1.609 (IL-6 4 h), and 0.2002 (IL-6 8 h). For 24 and 48 h, experiments were conducted independently and compared with a Student’s *t* test for which a *p* value < 0.05 was defined as significant

**Fig. 4:**
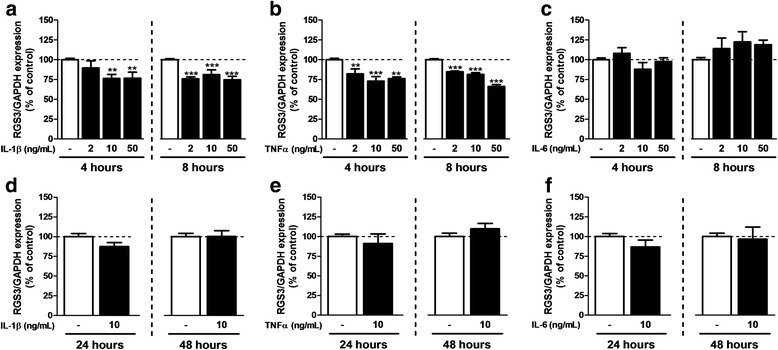
RGS3 expression after exposure to inflammatory cytokines. qPCR was performed on samples after 7 days in FBS 3%-medium in the presence of 2, 10, or 50 ng/mL of IL-1β (**a**, **d**), TNFα (**b**, **e**), and IL-6 (**c**, **f**). Cytokines were added during the 4, 8, 24, and 48 last hours of maturation. RGS3 mRNA expression was normalized to GAPDH expression. Each experiment was conducted independently. The FBS 3% condition was used as control. Data are presented as mean ± SEM; *n* = 6; ANOVA one-way with Dunnett’s post test; *p < 0.05, **p < 0.01 (vs control). *F*_3,30_ = 5.841 (IL-1β 4 h), 19.03 (IL-1β 8 h), 12.31 (TNFα 4 h), 66.60 (TNFα 8 h), 1.955 (IL-6 4 h), and 3.186 (IL-6 8 h). For 24 and 48 h, experiments were conducted independently and compared with a Student’s *t* test for which a *p* value < 0.05 was defined as significant

**Fig. 5:**
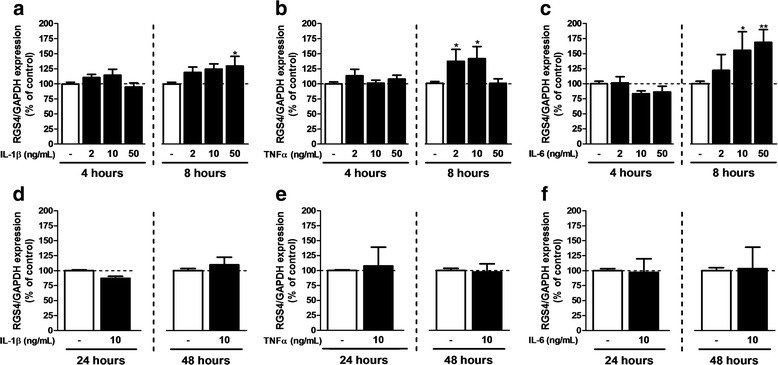
RGS4 expression after exposure to inflammatory cytokines. qPCR was performed on samples after 7 days in FBS 3%-medium in the presence of 2, 10, or 50 ng/mL of IL-1β (**a**, **d**), TNFα (**b**, **e**), and IL-6 (**c**, **f**). Cytokines were added during the 4, 8, 24, and 48 last hours of maturation. RGS4 mRNA expression was normalized to GAPDH expression. Each experiment was conducted independently. The FBS 3% condition was used as control. Data are presented as mean ± SEM; *n* = 6; ANOVA one-way with Dunnett’s post test; *p < 0.05, **p < 0.01 (vs control). *F*_3,30_ = 2.425 (IL-1β 4 h), 3.825 (IL-1β 8 h), 1.365 (TNFα 4 h), 4.134 (TNFα 8 h), 1.173 (IL-6 4 h), and 6.218 (IL-6 8 h). For 24 and 48 h, experiments were conducted independently and compared with a Student’s *t* test for which a *p* value <0.05 was defined as significant

**Fig. 6:**
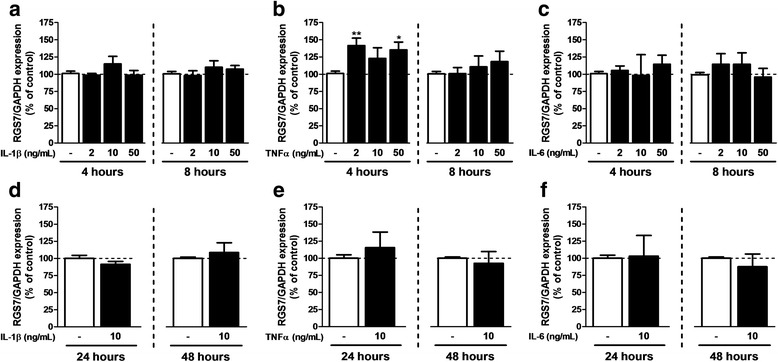
RGS7 expression after exposure to inflammatory cytokines. qPCR was performed on samples after 7 days in FBS 3%-medium in the presence of 2, 10, or 50 ng/mL of IL-1β (**a**, **d**), TNFα (**b**, **e**), and IL-6 (**c**, **f**). Cytokines were added during the 4, 8, 24, and 48 last hours of maturation. RGS7 mRNA expression was normalized to GAPDH. Each experiment was conducted independently. The FBS 3% condition was used as control. Data are presented as mean ± SEM; *n* = 6; ANOVA one-way with Dunnett’s post test; *p < 0.05, **p < 0.01 (vs control). *F*_3,30_ = 1.453 (IL-1β 4 h), 0.8085 (IL-1β 8 h), 5.515 (TNFα 4 h), 0.8064 (TNFα 8 h), 0.2283 (IL-6 4 h), and 0.9325 (IL-6 8 h). For 24 and 48 h, experiments were conducted independently and compared with a Student’s *t* test for which a *p* value <0.05 was defined as significant

Considering the documented inflammatory response that develops in the dorsal spinal cord of rodents undergoing peripheral nerve lesion [13, 33], we have specifically studied the impact of selected inflammatory cytokines on the expression of RGS in astrocyte cultures. As shown in Figs. 3 and 4, RGS2 and RGS3 mRNA were substantially decreased after exposure of astrocytes to either IL-1β, TNFα, or IL-6. A significant reduction in RGS2 expression was only observed after exposure to high concentration (50 ng/mL) of IL-1β and TNFα (Fig. 2a, b) or after longer exposure (24 h) to the three cytokines (Fig. 2d–f). Conversely, RGS3 underwent a rapid downregulation after 4 h exposure to TNFα and IL-1β (Fig. 4a, b) at almost all the concentrations tested whereas IL-6 did not significantly influence RGS3 expression in astrocytes. After longer exposure (24 and 48 h) to any of the three cytokines tested, no changes in RGS3 expression were observed. At variance with RGS2 and RGS3, the RGS4 expression was upregulated in astrocytes after exposure to an inflammatory environment (Fig. 5a–c). This was essentially validated after 8 h exposure to high concentrations of IL-1β (50 ng/mL) and IL-6 (10 and 50 ng/mL) and low concentrations of TNFα (2 and 10 ng/mL). Surprisingly, TNFα at the highest concentration did not evoke any modification of RGS4 expression as compared to the control (Fig. 5b). After 24- and 48-h exposure to inflammatory cytokines, RGS4 expression does not differ from control conditions. Finally, in the conditions tested, IL-1β and IL-6 exposure were without impact on RGS7 expression in astrocytes (Fig. 6a, c). In contrast, after 4 h of exposure to low concentration of TNFα, an increase in RGS7 mRNA expression was observed (Fig. 6b), which was however not maintained after 8 h. After 24 h of exposure, the level of expression was similar to the control conditions. For all the RGS, significant effects were generally dose-dependent and the longest exposure (48 h) to any inflammatory cytokines did not evoke any change, compared to control conditions. Noteworthy, RGS4 was the only RGS to be significantly affected by IL-6 exposure.

## Discussion

The present study shows that selected RGS expressed in cultured astrocytes are subjected to differential regulation in response to change in their chemical environment as well as in response to inflammatory stimuli. We herein also highlight a rapid regulation of these RGS in the spinal cord of animal undergoing SNI, a model of neuropathic pain in which neuroinflammation and sustained astrogliosis are documented [6, 38, 39]. Together with previous reports independently supporting the role of astrocytes and RGS in the modulation of neuropathic pain, we propose that the inflammation-driven modulation of RGS in astrocytes may participate in the pathological mechanisms that take place in neuroinflammatory contexts such as neuropathic pain.

Over the last two decades, many groups have reported on the putative role of RGS in neuropathic pain [26, 29, 40]. Several studies have examined the influence of manipulating either the expression or the activity of RGS using transgenic models or pharmacological inhibitors, respectively. Thereby, the functional interaction between RGS and receptors driving analgesic responses such as cannabinoid receptors [26] and opioid receptors [27] has been highlighted by evidencing increased analgesia after manipulation of RGS. Other groups showed that in RGS knockout-mice, pain relief mediated by opiates [27, 41] and antidepressants [31, 40] was modified, in particular after SNI. These observations support a role for RGS in the regulation of responses to both endogenous neurotransmitters and exogenous analgesics, especially in a context of neuropathic pain. Besides, there is accumulating evidence that the development of chronic pain in a variety of animal models is correlated with an alteration in the expression of RGS. Thus, after spinal cord injury, RGS7 expression was induced in spinal neurons and activated microglia [42]. In a model of partial sciatic nerve ligation, neuropathic pain was associated with an upregulation of RGS4 in the spinal cord [26], corroborating previous observations by Garnier et al. [43]. Other studies have reported on a downregulation of RGS3 and RGS4 in primary sensory neurons after transection of the sciatic nerve [29]. Taken together, this indicates that in different models sharing neuropathic pain features, RGS modulation can adopt distinct profiles, supporting the concept that diverse factors, among which neuroinflammation, can influence RGS expression. However, due to the lack of specific tools to detect RGS proteins and their relatively low endogenous expression [44], little is known so far about the cell-specific regulation of RGS in a neuropathic pain context and a large majority of these studies, including the present one, only examined the mRNA expression.

Herein, we used a model of standardized SNI that offers the advantage to elicit robust and substantial behavioral and molecular changes that closely mimic many features of clinical neuropathic pain [45]. We have examined the putative alterations in the expression of four RGS, selected on the basis of their nervous localization [24, 25] and the established interaction with GPCRs participating in pain processes, as described earlier. After validation of the SNI procedure by confirming mechanical allodynia in the ipsilateral paw, the animals were sacrificed at different time points, up to 3 weeks after the surgery to analyze the modifications of RGS during the early and late stages of the neuropathic model. Our data evidence a differential regulation of RGS with a downregulation of RGS2, RGS4, and RGS7 and an upregulation of RGS3, suggesting a differential control of their expression in this pathological context. As some of these RGS were proven to interact both with antinociceptive receptors [26, 27] and receptors that could play a pronociceptive role such as metabotropic glutamate receptor 5 [46], a complex modulation of these RGS could affect spinal GPCR function and either support or prevent the pain sensitization process. We herein also observed that RGS show distinct regulation profiles until 3 weeks after the lesion. Thus, RGS3 was only affected at early stages of the disease (3 and 5 days), suggesting a short-term role for this RGS. Other RGS, such as RGS2 and RGS4, were significantly modified at later time points, respectively 21 and 7 days. The transient regulations observed for RGS2, RGS3, and RGS4, either early or lately, could be explained by the fact that neuropathic pain is a dynamic process where changes occur in the spinal cord over days or weeks after the lesion in terms of cell infiltration and release of mediators [47–50]. Thereby, from 7 days after the lesion, combination of events that influence the expression of RGS3 may result in an expression level comparable to sham animals, whereas it is sufficient to elicit a change in RGS4 and later in RGS2 expression. Finally, RGS7 was modified at every time point studied, suggesting that this RGS could be concerned during both the initiation and maintenance phases of neuropathic process. While further observations with spatial and signaling-specific investigations of RGS modifications are needed, this identifies RGS as potential targets to interfere with neuropathic pain at different stages of the disease.

Several studies have highlighted the role of reactive glial cells in neuropathic pain but the expression of RGS in these cells remains so far poorly investigated [51, 52]. The expression of RGS in astrocytes and their regulation was herein examined in primary cultures exposed to selected conditions in which cells adopt typical characteristics of reactive astrocytes [53–56]. Thus, prolonged exposure of astrocytes to either dBcAMP or a defined supplement of growth factors (G5 supplement) induced their differentiation into phenotypes with distinct proliferation rates, morphologies, and cellular organizations. Moreover, we herein show that the choice of specific culture protocols, which differently maturate astrocytes, can influence the expression of RGS and thereby potentially impact on GPCR activity. This also confirms that individual astrocytic RGS undergo specific regulation in response to changes in their environment. Considering the selectivity of these proteins towards GPCRs [57, 58], an increase or decrease in RGS expression could silence or reinforce associated signals.

The development of neuropathic pain after SNI is associated with the release of inflammatory cytokines in the dorsal spinal cord that directly impact on astrocyte functions [13, 33]. Therefore, we herein modeled such pathological context by exposing cultured astrocytes to IL-1β, TNFα, or IL-6, given the essential role of these three cytokines in the development and maintenance of neuropathic pain [52, 59]. The regulation profiles differed in terms of kinetic as some RGS were modified at unique time points (RGS4 and RGS7) whereas others underwent more sustained modifications (RGS2 and RGS3). However, for all the RGS tested, prolonged exposure to inflammatory cytokines (48 h) did not influence significantly RGS expression, confirming that RGS are quickly regulated genes. It is noteworthy that the regulation of RGS in astrocytes exposed to these cytokines did not recapitulate the changes observed in the spinal cord of the animal undergoing SNI. This obviously reflects the complexity of the in vivo models where cells are exposed to a large set of mediators, including inflammatory cytokines, growth factors, and transmitters [5, 7] that could differentially regulate RGS expression in both neurons and glial cells. Consistent with this idea, we have observed that differential activation of astrocytes distinctly affected RGS expression. The modifications here observed probably result from different cell-specific regulation and may be controlled by diverse signaling pathways. Exposing cells in culture to selected cytokines constitutes a first step in clarifying the complex regulation that could operate in vivo. A next step should include a detailed study on individual cell types present in the spinal cord. With this research, we evidenced that RGS expression in astrocytes is influenced by changes in their environment, as tested in vitro by different activation media and cytokines, which are comparable to perturbations observed in a neuropathic pain context.

## Conclusions

Astrocytes express several receptors that sense the presence of neurotransmitters and neuromodulators [3, 60] and can further influence synaptic activity, particularly by the release of gliotransmitters [4]. Changes in the expression or activity of these receptors and their signaling partners inevitably affect the astrocyte responsiveness and synaptic plasticity. RGS proteins can modulate both the intensity of cell signaling as well as the coupling specificity of GPCRs [23]. Therefore, in the context of peripheral nerve lesions that trigger central neuroinflammation and astrocyte activation, their dynamic regulation could influence synaptic activity and participate in central sensitization [5]. Such regulation could be implicated in the genesis and the maintenance of neuropathic pain as changes in signal transduction, including nociceptive signals, can further lead to pain chronification [13, 61]. Considering the heterogeneity of processes supporting the development of neuropathic pain, many of which remain not fully elucidated and differ according to the lesion model [45, 62], a better understanding of the underlying molecular mechanisms should contribute to a better control of the disease. In this purpose, while little is known regarding their role and regulation in glial cells, our study identifies RGS as putative candidates for the modulation of astrocytic response in a context of neuroinflammation, a key feature of neuropathic conditions.

## Abbreviations

Ask1: Apoptosis signal-regulating kinase 1
CB1: Cannabinoid type-1 receptor
DAPI: 4′,6-Diamidino-2-phenylindole
dBcAMP: N^6^,2′-O-Dibutyryladenosine 3′,5′-cyclic monophosphate
FBS: Fetal bovine serum
GAPDH: Glyceraldehyde 3-phosphate dehydrogenase
GFAP: Glial fibrillary acidic protein
GPCR: G-protein-coupled receptor
IL-1β: Interleukin-1β
IL-6: Interleukin-6
JNK: c-Jun N-terminal kinases
PBS: Phosphate-buffered saline
PCR: Polymerase chain reaction
PWT: Paw withdrawal threshold
RGS: Regulator of G-protein signaling
SNI: Spared nerve injury
TNFα: Tumor necrosis factor α
